# Access to needles and syringes and methadone maintenance therapy among people who inject drugs in Yangon, Myanmar: a qualitative study

**DOI:** 10.1186/s12954-022-00692-8

**Published:** 2022-09-29

**Authors:** Win Lei Yee, Bridget Draper, Kyi Thar Myint, Win Min, Hla Htay, Daniel O’Keefe, Margaret Hellard

**Affiliations:** 1 Burnet Institute, Yangon, Myanmar; 2 Burnet Institute, Melbourne, Australia; 3 School of Public Health and Preventive Medicine, Monash University, Melbourne, Australia; 4 Hepatitis Service, Department of Infectious Diseases, Alfred Hospital, Melbourne, Australia; 5 Doherty Institute, Melbourne, Australia; 6 School of Population and Global Health, University of Melbourne, Melbourne, Australia

**Keywords:** People who inject drugs, Needles and syringes, Methadone

## Abstract

**Background:**

Access to sterile needles, syringes and methadone maintenance therapy (MMT) is critical to reduce the prevalence of bloodborne virus infections among people who inject drugs (PWID). We aimed to explore the experiences of PWID with respect to accessing needles/syringes services and MMT in Yangon, Myanmar.

**Methods:**

Burnet Institute implemented a community-based hepatitis C testing and treatment (CT2) program for PWID with on-site needles and syringes distribution. Separate from CT2, MMT was available at two government-run sites in Yangon. We conducted in-depth interviews with 15 PWID who received hepatitis C care in this program. Interviews were transcribed verbatim and translated into English. Thematic data analysis was performed using NVivo12 software.

**Results:**

Self-reported changes to needles/syringes sharing behaviour after hepatitis C education in the CT2 program and commencement of treatment were observed. One third of participants reported they became aware of the risks of sharing and reusing needles/syringes, and consequently refrained from sharing after the CT2 program. Inadequate availability of NSPs, cost of needles/syringes, and issues maintaining privacy when accessing needles/syringes emerged as key barriers to accessibility of needles/syringes. Participants described difficulties in accessing free needles/syringes. They were not aware of other free needles/syringes services at the time of the interview. Purchasing needles/syringes from pharmacies had privacy and confidentiality concerns. Structural barriers to accessibility of MMT were identified for both MMT sites in Yangon. Of the two MMT sites in Yangon, participants reported that the Ywarthargyi center had strict eligibility criteria for take-home methadone and transportation issues as it was located in the outskirt of the town. The Thingyangyun center was in a more convenient location, but only offered daily observed doses and had a long waiting time which was burdensome for some employed participants.

**Conclusion:**

Expansion of free needles/syringes services and adaptations of MMT to consider the needs and individual preferences of PWID will improve their access to these services and would likely reduce injecting related harms.

## Background

Globally, the prevalence of bloodborne virus (BBV) infections is high among people who inject drugs (PWID). In 2019, approximately 13% of PWID were living with HIV, 50% were hepatitis C virus (HCV) antibody positive, and 9% were hepatitis B surface antigen positive (indicating current infection) [1]. Needle and syringe programs (NSP) and methadone maintenance therapy (MMT) are important tools for preventing BBV infections. Evidence shows that the access to sterile needles/syringes can effectively reduce HIV and HCV infections, and with high coverage, the effectiveness is similar between low- and middle-income countries and high-income countries [2, 3]. Combination of the NSP and MMT use can further reduce HCV infections among PWID [4].

In Myanmar, BBV infections among PWID mirror the global epidemiology. Estimates from 2018 found that HCV antibody prevalence among PWID was 56%, HIV prevalence 19%, and HBV prevalence 7.7% [5]. Access to sterile needles and syringes is mainly through NSPs operated by non-governmental organizations (NGO), which provide free needles/syringes; in addition, pharmacies sell needles/syringes. However, access to sterile injecting equipment across Myanmar is inconsistent and inadequate. As a result, reports of receptive needle/syringe sharing are common, with 7–63% of PWID reporting the practice, depending on location [5]. In Yangon, the largest city in Myanmar, 36% of respondents reported receptive sharing in 2017–2018 [5]. Research in neighbouring countries such as Thailand and China, has found rates of sharing syringes of 18–31% [6–8]. Few studies have investigated access and barriers to needles/syringe services in Myanmar.

Methadone maintenance treatment, an opioid agonist therapy option, has been available in Myanmar since 2006. There are two methadone treatment centers in Yangon: Ywarthargyi Mental Health Hospital (hereafter, Ywarthargyi), which provides take-home methadone to stable patients, and Thingangyun General Hospital (Thingangyun), which provides directly observed daily doses and (from early 2020, due to COVID-19 restrictions) take-home doses. An evaluation of the MMT program conducted in 2013 found the operational hours, location, registration process and confidentiality of these centers hampered treatment access for many patients [9, 10]. No other studies have explored the accessibility and acceptability of the MMT program in Myanmar. Further evidence is needed to understand the experiences of PWID taking MMT to inform program implementation.

In 2019, Burnet Institute researchers implemented a community-based HCV testing and treatment (CT2) study at two community clinics in Yangon, Myanmar; one clinic included an on-site NSP. At the end of the study, we conducted a qualitative sub-study with participants to explore their drug use behaviour, experiences of accessing harm reduction services, and the acceptability of the community-based clinic. This manuscript reported the results of that sub-study, describing the experiences of PWID with respect to MMT and NSP access in Yangon.

## Methods

The study design, methods and outcomes of the CT2 study are described elsewhere [11, 12]. Briefly, Burnet Institute’s clinic, one of the two CT2 study clinics, provided point-of-care HCV testing and treatment to PWID, including on-site distribution of sterile needles and syringes. The community-based CT2 clinic was located near the public Thingangyun methadone treatment center. At the CT2 clinic, PWID received pre- and post-test counselling, followed by anti-HCV antibody testing and viral load testing if they tested anti-HCV antibody positive. Those who tested positive for HCV viral load were assessed and treated with direct-acting antivirals if appropriate.

In November 2019, we conducted in-depth interviews with 15 PWID who received HCV testing and treatment via the CT2 study. We recruited participants using purposive sampling to ensure a mix of genders, ages (18 years or over), injecting behaviours and achievement of sustained virologic response (cure) post-treatment. The interviews were conducted face-to-face using a semi-structured interview guide. The interview guide explored participant demographics, HCV testing and treatment experience at the clinic, access to and acceptance of the service, and drug use behaviour. Participants were asked about changes to their use of sterile needles and syringes after treatment, which led to discussion about access to NSPs, and those on MMT were asked about their experience of the treatment program. Written informed consent was obtained from all participants before the interview. The interviews were audio-recorded and averaged about 60 min. Data collection for qualitative interviews continued until response saturation was reached.

The first author, who has experience in qualitative research, undertook the interviews and thematic data analysis. The interviews were transcribed verbatim and translated into English. The transcripts were analysed in NVivo12 (QSR International, 2018), applying inductive analysis to investigate the participants’ experiences of NSPs and MMT. Text data were coded and categorized into groups which were then framed into different themes. Emerging themes were reviewed by the second author to reframe and finalize the themes. Data were cross-checked with interview notes of the researcher to ensure data accuracy and validity.

## Results

Of our 15 participants, two were women and 13 were men; their average age was 33 years, and all were residents of Yangon. Six of them were unemployed and thirteen were currently receiving MMT. Twelve participants reported that they contracted HCV infection from sharing needles/syringes or injecting equipment, one from sexual contact and the other two did not know the source of infection.

Three themes emerged from the data analysis related to use of needles and syringes and MMT: (1) self-reported changes in needle and syringe sharing behaviour post-HCV education and treatment, (2) barriers to accessing needles and syringes and (3) barriers to accessing methadone maintenance therapy.

### Self-reported changes in needle and syringe sharing behaviours

Needle and syringe sharing behaviour was common among the participants, particularly when there was inadequate availability of needles/syringes, but some reported changes in behaviour after participating in the CT2 program. Participants reported sharing and repeatedly reusing needles/syringes among groups of peers who inject drugs before their involvement in CT2. About one third of participants said they became aware of the risks of needle sharing after learning their HCV status and participating in the CT2 program. They reported that following treatment, they refrained from sharing needles and other equipment.

I didn’t care much about other injecting equipment previously, but only syringes … now I am using my own sterile water vial and my own dissolving cup. I try not to share them with others. (BI08)

When a group of peers occasionally had insufficient needles/syringes to inject drugs separately, participants reported use of other risk reduction strategies instead. Each peer in the group disclosed their HCV status to let others decide whether to share with them.

### Barriers to accessing needle and syringe

Participants reported that there were two options for accessing needles/syringes—NGO-led NSP which distributed needles/syringes to PWID free-of-charge and private pharmacies through payment for needles/syringes. Participants reported there being few NGO-led, free NSP options in Yangon and described difficulties in purchasing needles/syringes from pharmacies.

#### NGO-led NSPs

Many participants said that the CT2 NSP was the only free service available in Yangon; they were unaware of any other free NSP services at the time of interview.

I take from Burnet clinic. There are four syringes per packet … it is convenient to use … I can buy it from the pharmacy but not from every pharmacy ... I knew only this Burnet [needle and syringe program] I don’t even know if there are other organizations. (BI04)

One participant mentioned difficulty in acquiring free needles/syringes when Burnet Institute stopped a previous harm reduction project.

Burnet Institute used to distribute sterile needles and syringes before. There is a gap between old project and the current project, and we had many difficulties in getting needles and syringes during this interval [two years]. (BI01)

#### Private pharmacies

The participants faced challenges in purchasing needles/syringes from private pharmacies when they could not access a free NSP. Needles/syringes were normally available at many pharmacies but, because of legal concerns, some pharmacies will not sell them to customers suspected of using illicit drugs. Participants also raised privacy and confidentiality concerns related to purchasing needles/syringes from local pharmacies.

When we buy needles, they usually see us as drug addicts. I’m afraid the pharmacy owner may inform the police or if my parents visit the pharmacy, he might have told them that I had bought needles from him. (BI01)

As a result, participants described travelling to other pharmacies to purchase needles/syringes. They chose pharmacies they knew would sell them the brand they liked, sometimes located in townships far away from their homes. Prices ranged from 200 to 1500 kyat (USD 0.15–1) per needle and syringe.

### Barriers to accessing methadone maintenance therapy and views of take-home dosing

Among the 13 participants receiving MMT, eight received daily observed doses at Thingangyun and five received weekly take-home methadone from Ywarthargyi. The participants shared their experiences of accessing the methadone program and the challenges encountered. Long queues and daily visits to the Thingangyun center were burdensome for the participants taking daily observed doses at Thingangyun. On the other hand, transportation difficulties and strict criteria of the Ywarthargyi center deterred some participants who preferred take-home methadone from attending.

#### Barriers to accessing Thingangyun daily dosing methadone clinic

The Thingangyun clinic provided daily doses at 7:30 every morning, but a long queue meant those who were employed needed to arrive as early as 7:00 am. One participant reported concern about his methadone use being revealed to colleagues, based on his past experience of workplace discrimination.

I finish taking methadone as early as 8 am as there is a long queue at the methadone clinic which opens at 7:30 am. If the people from work also find out that I am a methadone client, they would discriminate against me … It has happened before and then I didn’t want to work there anymore. (BI01)

#### Barriers to accessing Ywarthargyi take-home methadone clinic

Participants said Ywarthargyi’s take-home methadone program applied strict criteria, including urine and psychological test results, a physician’s approval and accompaniment of a family member.

Some participants who preferred take-home dosing had to continue with daily observed dosing at Thingangyun because it was more convenient than attending Ywarthargyi. To access take-home doses at Ywarthargi, clients had to attend on weekdays to receive take-home doses. Given Ywarthargi is on the outskirts of Yangon, transport was also an issue for some. These accessibility barriers made attending work more difficult.

I take a bus from Sanchaung to Dagon University first, then I take a different bus from Dagon University to Ywarthargyi Hospital … It usually takes 3 to 4 hours … I couldn’t work on that day. (BI09)

When comparing the two options, one female participant explained that daily visits to the methadone center encouraged drug use by creating an environment to meet peers and facilitating access to drugs. She believed that take-home dosing would improve her adherence and reduce her drug use.

I usually meet many peer friends here [methadone clinic]. I can’t resist the persuasion of my friends and the sight of drugs. When I have money, I end up using drugs with friends. If I get take-home methadone dosage, I’d spend more time at home and would not have to see my friends as usual. (BI02)

#### Views and experiences of take-home methadone programs

The participants had different views on the market for methadone diversion. One participant reported that Ywarthargyi's strict rules for take-home methadone dosing were designed to combat methadone diversion. She claimed some users liked to stockpile doses in case they were unable to access the methadone center.

There are few guys who sell their take-home methadone medications. Because of them, we are denied to take-home methadone dosage… Some people stored a few doses of methadone at their home in case of an emergency. They will need to take methadone at home when they could not go to the hospital to take daily methadone administration. (BI02)

Another participant who was an occasional illicit methadone user related a different experience. He was concerned about addiction resulting from regular doses from the methadone program, so instead took the surplus methadone of his friends in exchange for money or food.

Their methadone dosage is 7 ml (70 mg), for instance. So, they limit their daily methadone intake themselves by taking 5 ml and don’t take the remaining 2 ml ... I took their surplus methadone by giving them money or treating them to snacks. (BI13)

## Discussion

Our study illustrated the importance of engaging PWID in care and providing education on HCV and other BBVs to improve uptake of harm reduction strategies. We also identified numerous barriers to accessing harm reduction services in Yangon, including limited availability of NSPs and barriers to accessing available services, and barriers to accessing MMT services.

Our study found that PWID in Yangon had few options for obtaining needles/syringes. Until the Burnet NSP reopened, their main option was to purchase injecting equipment from pharmacies. This is consistent with the findings of a nationwide survey conducted in 2018, which showed that participants from Yangon had the lowest use of sterile injection equipment, and more than half reported pharmacies as their main source of needles/syringes [5]. However, we found that purchasing needles/syringes from pharmacies was unpopular; aside from the cost, many participants described a sense of insecurity when doing so. Participants reported concerns about privacy and confidentiality at pharmacies, but also fear of arrest and incarceration after identification as a person who uses drugs. Like in many settings, law enforcement and criminalization of drug use in Myanmar impedes access to harm reduction services including NSP and MMT [8, 13].

A study conducted in Myanmar in 2017 reported 19% of PWID experienced insufficient needle/syringe coverage [14]. This was prior to the closure of NSPs funded by Three Millennium Development Goal Fund (3MDG) in 2017, which meant Yangon went a year without a formal NSP. Low coverage of NSP is associated with prevalent and frequent needle/syringe sharing among PWID, placing them at risk of BBV infections [15]. Expansion of NSP for distribution of sterile needles/syringes at no cost in Yangon and decriminalization of drug use are imperative to improve the needle/syringe access of PWID.

Our study found that PWID in Yangon faced multiple structural barriers in accessing MMT programs. In 2019, there were 55 MMT centers in Myanmar but only two in Yangon [16]. These two centers had different procedures for dispensing methadone, each with advantages and disadvantages. Thingangyun is located centrally, and our research participants appreciated the consequent short travelling time and low transportation costs. Its disadvantages were daily visits and long queues to receive methadone, a substantial access barrier for employed clients. In contrast, Ywarthargyi provided take-home doses requiring a single weekly visit, but its distant location and weekend closure reduced access for clients which also restricted access to for employed clients. Participants’ methadone dispensation preferences were therefore influenced by their employment status; some could not switch to take-home methadone due to the demands of travel and work. The Ywarthargyi center also imposed as strict eligibility criteria, which may deter some PWID from switching to take-home doses.

Myanmar has low coverage of opioid agonist therapy, with 16,348 PWID (17% of the total estimated population) receiving methadone in 2019 [16]. For effective prevention of HIV among PWID, according to the WHO, UNODC and UNAIDS guidelines (2009), opioid agonist therapy coverage should be above 40% [17]. Following the Myanmar COVID-19 epidemic, stay-at-home orders and movement restrictions resulted in many MMT centers changing policy to allow take-home dispensation. Patients were given two weeks of take-home doses at the beginning of the pandemic, increasing to a month’s supply at the pandemic’s peak; the program reverted to bi-weekly dispensation in early 2022 as COVID-19 cases declined. Service users and providers with whom the researchers were in contact did not report increases in overdoses or black-market availability of methadone during this time; however, this data was anecdotal, and was not part of the qualitative sub-study (conducted before the COVID-19 epidemic in Myanmar).

Adopting a more flexible approach to prescribing and dispensing methadone doses would improve engagement and retention in MMT. Service delivery modifications could include weekday and weekend pick-ups, shorter wait times, and more flexibility to switch between take-home and daily dosing at both Thingangyun and Ywarthargyi, especially for employed patients.

## Conclusion

Our findings highlight the need for increased access to harm reduction services in Yangon. Expansion of free NSP and MMT programs, with flexible dispensation policies that suit clients’ needs, will improve access to these services, thereby contributing to the prevention of BBV infections and subsequent harms.

## Data Availability

The dataset used in this study is available from the corresponding author upon reasonable request.
